# Supplementary material for: Doxorubicin- and Trastuzumab-Modified Gold Nanoparticles as Potential Multimodal Agents for Targeted Therapy of HER2+ Cancers
Source: Molecules. 2023 Mar 7;28(6):2451. doi: 10.3390/molecules28062451 (PMC10058186; doi:10.3390/molecules28062451)
Supplement: Supplementary file 1 [file molecules-28-02451-s001.zip › molecules-2199887-supplementary.pdf]

Supplementary Material

Doxorubicin- and Trastuzumab-Modified Gold Nanoparticles as Potential Multimodal Agents for Targeted Therapy of HER2+ Cancers

Kinga Żelechowska-Matysiak [1], Kamil Wawrowicz [1], Mateusz Wierzbicki [2], Tadeusz Budlewski [3], Aleksander Bilewicz [1] and Agnieszka Majkowska-Pilip [1,3,*]

[1] Centre of Radiochemistry and Nuclear Chemistry, Institute of Nuclear Chemistry and Technology, 03-195 Warsaw, Poland

[2] Department of Nanobiotechnology, Institute of Biology, Warsaw University of Life Sciences, 02-787 Warsaw, Poland

[3] Isotope Therapy Department, Central Clinical Hospital of the Ministry of Interior and Administration, 02-507 Warsaw, Poland

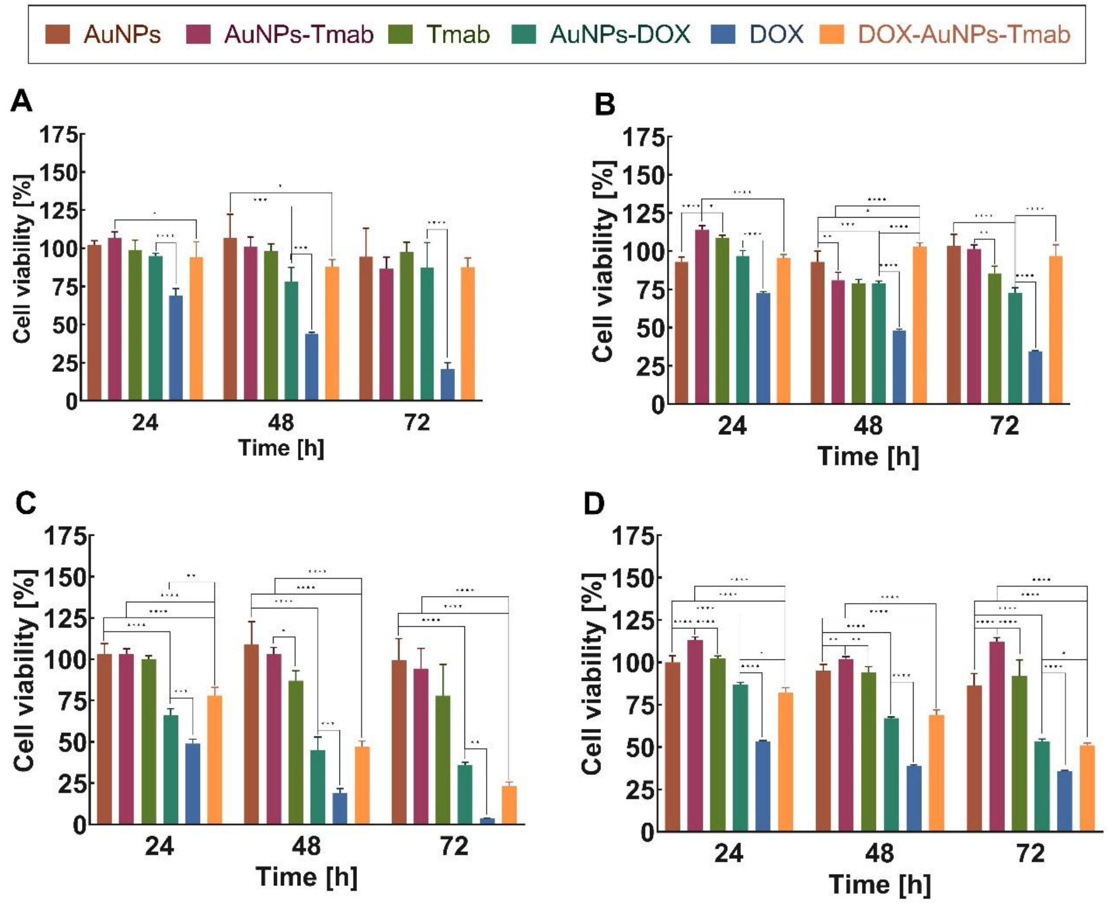

Figure S1. Metabolic activity of SKOV-3 (**A**, **C**) and MDA-MB-231 (**B**, **D**) cells after treatment with AuNPs, AuNPs-Tmab, Tmab, AuNPs-DOX, DOX, DOX-AuNPs-Tmab. DOX concentrations are 2 μg/mL (**A**, **B**) and 15 μg/mL (**C**, **D**). Cells were incubated for 24 h, 48 h and 72 h and then the MTS assay was performed. Untreated cells were used as control (100% viability). Statistics were performed using one-way ANOVA test comparing AuNPs together with nanoparticles with doxorubicin and/or trastuzumab attached, and AuNPs-Tmab was compared with Tmab/DOX-AuNPs-Tmab and AuNPs-DOX vs. DOX/DOX-AuNPs-Tmab. Summarized data represented results from four replicates (n = 4; mean ± SD) and were considered significant if $p \leq 0.01$ (**), $p \leq 0.001$ (***), $p \leq 0.0001$ (****).

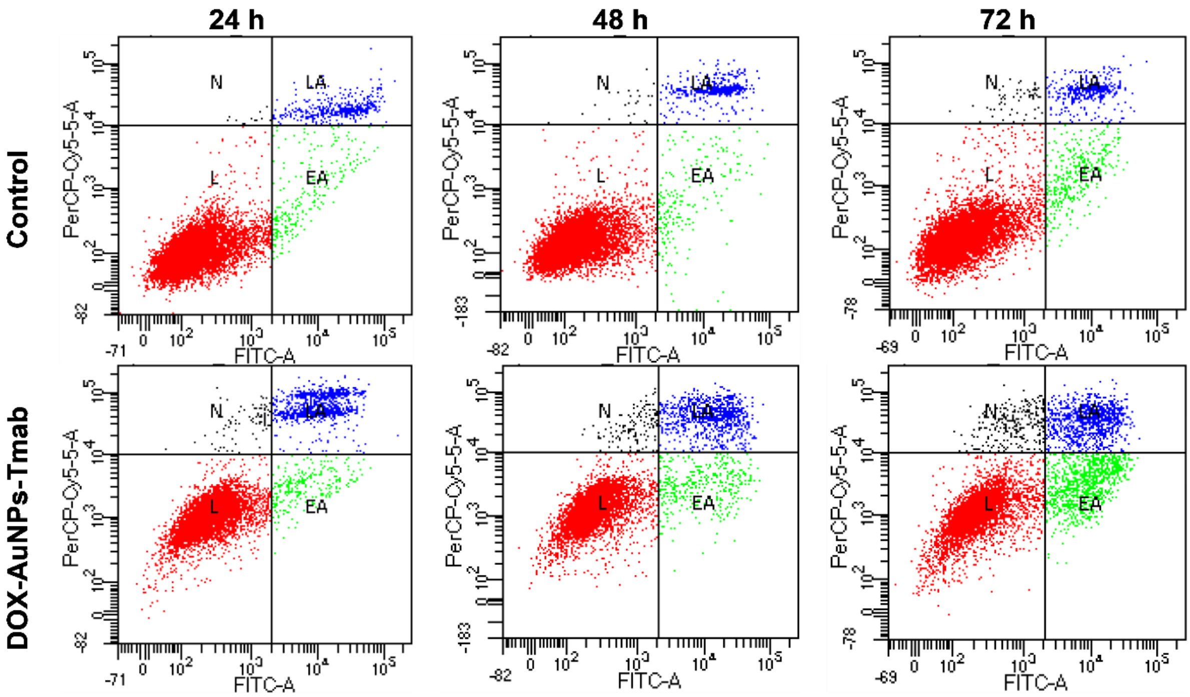

Figure S2. Representative graphs of apoptosis analysis results for the control group and for cells treated with DOX-AuNPs-Tmab after 24 h, 48 h and 72 h. DOX concentration is 7 µg/mL.

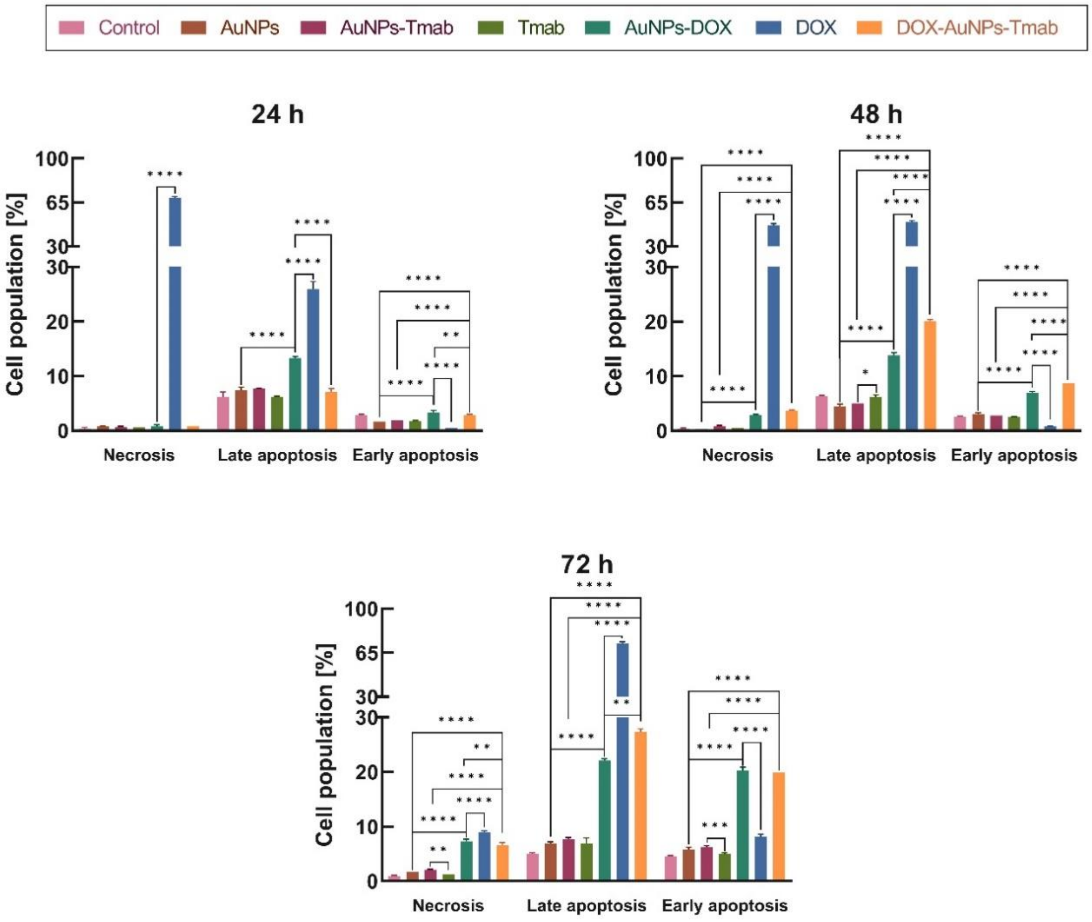

Figure S3. Distribution of SKOV-3 cell populations (necrosis, late and early apoptosis) treated with AuNPs, AuNPs-Tmab, Tmab, AuNPs-DOX, DOX, and DOX-AuNPs-Tmab after 24 h, 48 h and 72 h.

Data are presented from four replicates (n = 4, mean ± SD). DOX concentration is 15 μg/mL. Untreated cells were used as control, while statistics (one-way ANOVA test) were performed similarly as in the case of cytotoxicity studies with the use of MTS assay (Figure S1). Statistical significance was considered significant if $p \leq 0.05$ (*), $p \leq 0.01$ (**), $p \leq 0.001$ (***), $p \leq 0.0001$ (****).

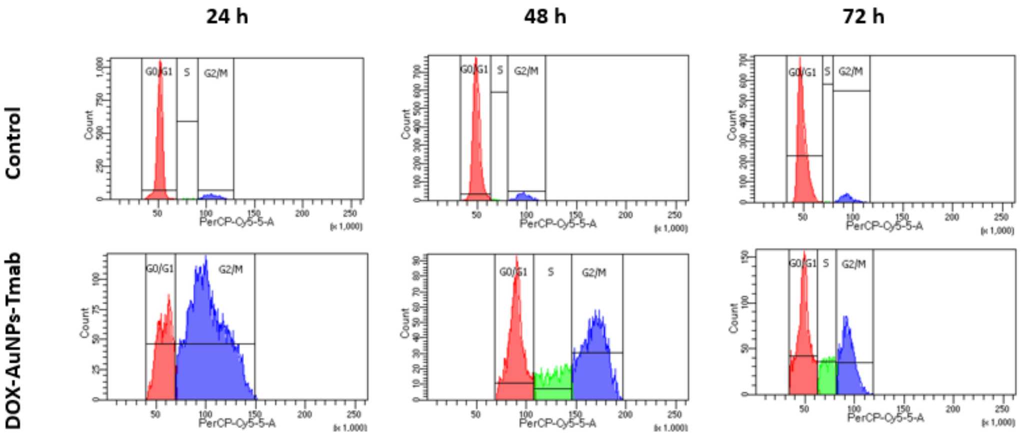

Figure S4. Representative graphs of cell cycle analysis results for the control group and DOX-AuNPs-Tmab cell treatment after 24 h, 48 h and 72 h. DOX concentration is 7 μg/mL.

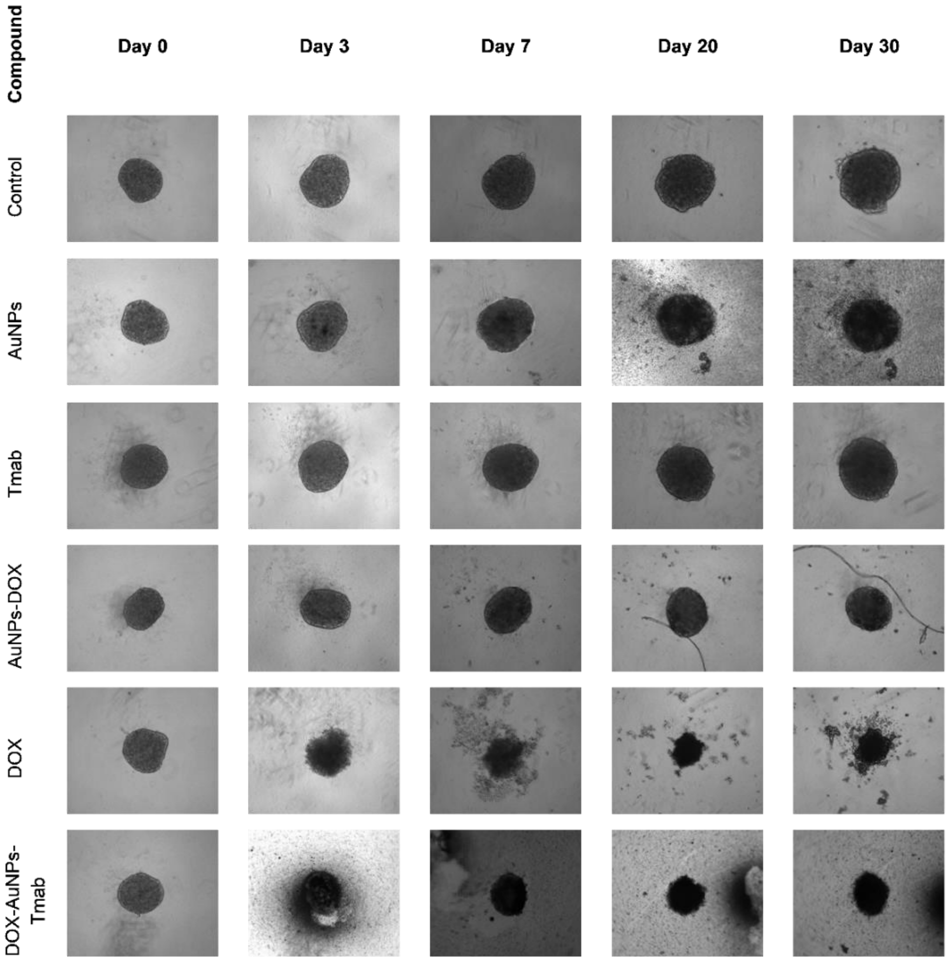

Figure S5. Microscopic images showing the morphology of control and compound-treated SKOV-3 spheroids on several selected measurement days.
